# Navigating Complications in Cardiac Pacemakers: A Comprehensive Review and Management Strategies

**DOI:** 10.31083/j.rcm2508299

**Published:** 2024-08-21

**Authors:** Anil Sriramoju, Shruti Krishna Iyengar, Komandoor Srivathsan

**Affiliations:** ^1^The Division of Cardiovascular Diseases, Mayo Clinic Hospital, Phoenix, AZ 85054, USA

**Keywords:** hemothorax, hematoma, CIED, pacemaker, thrombosis

## Abstract

The landscape of cardiac pacemaker technology has undergone significant evolution over the last two decades, transitioning from simple single-chamber devices to sophisticated multi-chamber rate-responsive systems and cardioverter defibrillators. This progression has introduced a complex array of complications inherent to device implantation and operation, encompassing both mechanical and clinical challenges. These complications notably include lead dislodgment, device migration, venous thrombosis, and hemothorax, which not only affect patient outcomes but also impose substantial economic burdens. This review meticulously analyzes these complications, elucidating their mechanisms, clinical implications, and the economic consequences associated with their management. It also outlines current and emerging strategies aimed at mitigating these complications, emphasizing the need for continual updates in clinical practices and protocols. Through this discourse, the review seeks to equip clinicians with a comprehensive understanding of these complications, thereby enhancing the safety and efficacy of cardiac pacing interventions.

## 1. Introductions

Over the past two decades, there has been a notable increase in the implantation of pacemakers (PPMs, permanent pacemakers), coupled with ongoing innovation in design and technology. These advancements have led to systems that are increasingly complex, evolving from single-chamber fixed-rate pacemakers to include multi-chamber, rate-responsive pacemakers capable of cardioversion, defibrillation (ICDs, implantable cardioverter-defibrillators), and/or cardiac resynchronization therapy (CRT). Cardiac implantable electronic devices (CIEDs) help analyze and regulate cardiac rate and rhythm (Table 1).

**Table 1.** **Pacemaker complications**.

Category	Complication	Description
Venous access related complications	Pneumothorax	Occurs when air enters the pleural space, potentially causing lung collapse.
	Hemothorax	Accumulation of blood in the pleural cavity, which can compress the lung and impair breathing.
	Pulmonary air embolism	Air bubbles enter the bloodstream and block pulmonary arteries, potentially life, threatening.
Lead related complications	Cardiac perforation	The lead punctures the heart wall, potentially causing cardiac tamponade or other serious issues.
	Infection	Infections at the site of the device implantation, which can spread and become systemic.
	Lead dislodgment	Movement of the lead from its original position, potentially causing the device to malfunction.
	Venous thrombosis	Formation of a blood clot within a vein, which can impede blood flow and cause swelling and pain.
	Conduction fracture	Breakage of the lead, which can interrupt the pacing or defibrillation functionality.
	Pacemaker exit block	Failure of the electrical impulse to exit the pacemaker lead, resulting in loss of pacing function.
	Insulation failure	Damage to the lead’s insulation, potentially causing short, circuiting or inappropriate shocks.
	Connection problem	Issues with the connections between the lead and the device, possibly causing malfunction.
Generator failure	Battery depletion	The battery runs out of charge, necessitating replacement to continue device function.
	Device migration	Movement of the device from its original position, which may require repositioning or replacement.
	Electromagnetic interference	Interference from external electronic devices that can alter pacemaker function.
	Trauma	Physical damage to the device or lead from external impacts or accidents.
	Radiation	Exposure to radiation which can alter device function or damage its components.

Conventional transvenous pacemakers primarily constitute a subcutaneous pulse generator and transvenous lead which are the primary culprits behind complications arising from these devices. To address these challenges, the concept of leadless pacemakers emerged. Leadless intracardiac pacemakers, deployed via a minimally invasive femoral vein approach, are entirely implanted within the right ventricle and are currently utilized in patients requiring single-chamber ventricular pacing.

## 2. Evolving Challenges and Economic Impact of Pacemaker Complications

The comparative analysis of complication rates in pacemaker implantations through the Mode Selection Trial (MOST) [1] and the FOLLOWPACE trial [2] provides important insights into the challenges of cardiac pacing, both conducted in the first decade of the 2000s. MOST, which focused on a select cohort within academic centers, reported a 4.8% complication rate post-implantation. In contrast, FOLLOWPACE, involving a broader patient base in varied hospital settings, had a higher acute complication rate of 12.4%, with 4.2% necessitating surgical intervention. These trials demonstrate how clinical settings and operator expertise significantly influence outcomes. Recent studies, like one by Cantillon *et al*. [3], indicate even higher complication rates, associated with the complex medical profiles of pacemaker recipients, including prevalent conditions like hypertension, diabetes, and coronary artery disease. This historical data serves as a foundation for discussing current advancements in pacemaker technology and patient management.

The shifting pattern of complications—from lead dislodgements and thoracic injuries towards electrical and mechanical issues within the pacing systems—highlights the dynamic nature of challenges in pacemaker management. This evolution calls for continuous updates in clinical practices and monitoring protocols to better cater to the changing needs of the patient population.

Additionally, the financial implications of pacemaker complications are profound. Issues related to pacemaker leads can impose costs akin to those of new pacemaker implantations, while more severe complications such as pericardial effusion can result in expenses comparable to multiple implantations. These economic considerations necessitate a broader financial analysis involving multiple stakeholders to fully understand and address the economic impact of pacemaker-related complications.

In this review, we will explore common complications such as Infection, hematoma/bleeding, and procedural mechanical issues that may adversely affect patients following pacemaker implantation (Table 2).

**Table 2.** **Types of pacemakers**.

Type of CIED	Description	Associated complications
Single chamber pacemaker	Stimulates either the right atrium or right ventricle.	Infection, lead dislodgment, lead fracture, device migration, electromagnetic interference.
Dual chamber pacemaker	Stimulates both the right atrium and right ventricle, allowing for coordinated pacing.	Infection, lead dislodgment, lead fracture, pacemaker syndrome, electromagnetic interference.
Implantable cardioverter-defibrillators (ICDs)	Can perform pacing and deliver shocks to correct life, threatening arrhythmias.	Infection, inappropriate shocks, lead fracture, device migration, electromagnetic interference.
Biventricular pacemakers (CRT)	Stimulates the left and right ventricles simultaneously to improve heart efficiency, mainly in heart failure patients.	Infection, lead dislodgment, battery depletion, venous thrombosis, electromagnetic interference.
Leadless pacemakers	Miniaturized pacemakers implanted directly in the heart, no leads required.	Device dislodgment, infection, limited battery life, retrieval issues.
Implantable cardiac monitors	Continuously record the heart’s electrical activity to diagnose arrhythmias, without pacing or defibrillation capabilities.	Infection, device migration, data transmission issues, skin irritation.

CIED, cardiac implantable electronic device; CRT, cardiac resynchronization therapy.

## 3. Access Related Complication

### 3.1 Pneumothorax

**Fig. 1.**
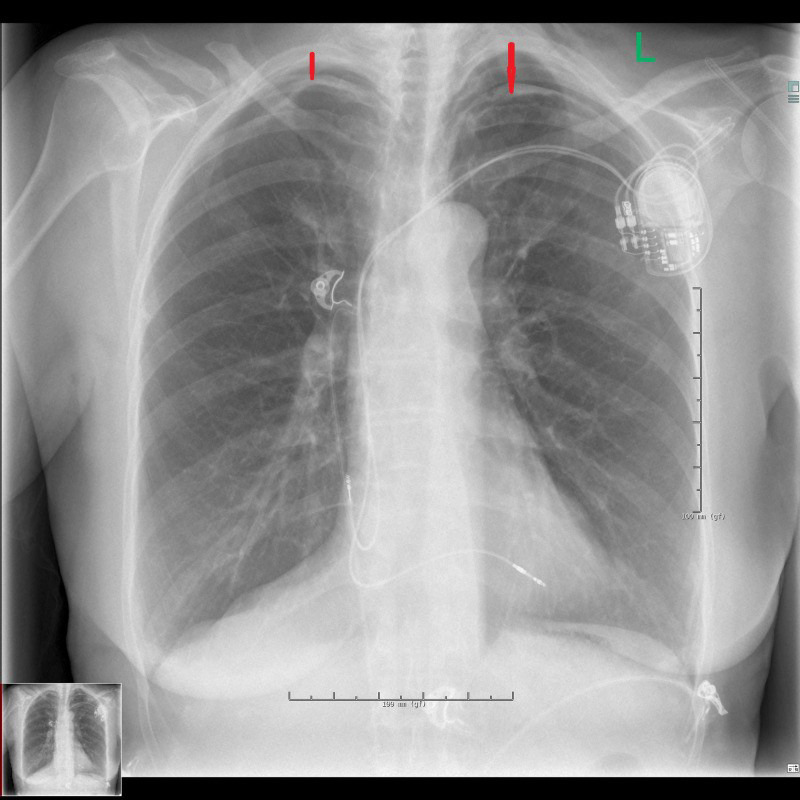
**Chest X-ray shows bilateral apical pneumothorax (red arrows) 3 hours after implantation of a pacemaker**. Olesen, L.L. (2020). [Image]. Creative Commons Attribution License. Retrieved from https://www.ncbi.nlm.nih.gov/pmc/articles/PMC7716385/[4].

Pneumothorax, characterized by the presence of air in the pleural space, can occur spontaneously or because of medical procedures, such as the insertion of CIEDs (Fig. 1, Ref. [4]). This complication, though often asymptomatic and incidentally detected on routine chest radiographs, warrants attention, especially in patients experiencing dyspnea and pleuritic chest pain post-CIED implantation.

Selecting the appropriate imaging modality for diagnosing pneumothorax depends on the stability of the patient’s presentation. Options include X-ray, computed tomography (CT), or ultrasound. Despite its generally low risk, pneumothorax can lead to increased morbidity, prolonged hospital stays, and in rare cases, mortality.

The frequency of clinically significant pneumothorax requiring intervention, such as chest tube placement, during pacemaker or cardiac device implantation procedures varies across studies. In a Danish nationwide cohort study of 28,860 patients, the overall incidence of pneumothorax requiring chest tube drainage was 0.66% [5]. In study by Ogunbayo *et al*. [6], analysis showed that in CIED procedures, pneumothorax (PTX) occurred with an incidence rate of 1.3%. The incidence of PTX peaked at 1.6% during 2012 and 2013. This increase may have been influenced by a shift from inpatient to outpatient CIED procedures. PTX was significantly associated with increased pulmonary complications, the necessity for chest tube insertion, extended hospital stays, and elevated healthcare costs. Risk factors for PTX included being over 80 years old, female gender, Caucasian race, existing chronic obstructive pulmonary disease, and the use of a dual-chamber device as opposed to a single-chamber device. The insertion of a chest tube was identified as a key determinant of worse outcomes and increased costs associated with PTX. Achieving transvenous vascular access for CIED procedures is crucial but poses risks, particularly due to the proximity of vital organs and vascular structures, such as the apex of the lung.

Techniques for venous access commonly involve:

(1) Axillary vein puncture.

(2) Cephalic vein cut down.

(3) Subclavian Vein Puncture.

Both subclavian vein puncture and cephalic vein cutdown are commonly used worldwide. However, cephalic vein cutdown is often considered time-consuming due to its technical nature and the requirement for specialized ultrasound for other access methods, which may not always be readily available [7].

Conventionally, subclavian, or axillary vein access is obtained using fluoroscopy-guided puncture, sometimes augmented by contrast venography, especially if the first pass puncture is unsuccessful or for subsequent lead insertion. This technique is fast and boasts a high success rate. Nonetheless, the rate of pneumothorax with this method is typically higher, around 1%, compared to the other two techniques.

Cephalic vein cutdown, while associated with a lower risk of pneumothorax, poses challenges due to the difficulty in cannulating the cephalic vein. Despite this, cephalic vein cutdown is increasingly being recommended as the primary method for venous access, with subclavian vein puncture as a backup.

Axillary vein puncture is another access method that may offer the benefits of both cephalic vein cutdown and subclavian vein puncture, as the axillary vein is extrathoracic in course. However, this technique has limited success in patients with deep veins and those who are morbidly obese.

Morton *et al*. [8] conducted a large, single-center retrospective analysis describing the use of a caudal 40° angulation instead of conventional antero-posterior (AP) fluoroscopy to guide subclavian vein puncture for the insertion of transvenous pacing wires for CIEDs. Their study demonstrated a reduction in pneumothorax rates and the use of contrast venography. Similar findings were reported by Yang *et al*. [9], has been shown to reduce pneumothorax rates as this technique provides the operator with a better understanding of needle depth in relation to important structures such as the pleural space, thereby improving safety. Specifically, the oblique view afforded by this technique allows for a better appreciation of the anterior surface of the lung and greater separation between the clavicle and the first rib, the space through which the puncture needle is advanced to cannulate the subclavian vein [10].

Ultrasound-guided access is also becoming increasingly common, minimizing the risk of pneumothorax. Ultrasound probes have been designed specifically for this purpose with a hockey stick-like shape that can be inserted into the pacemaker pocket to localize the course of axillary and subclavian veins. The course of the needle could be tracked on the ultrasound before entering the vein.

### 3.2 Hemothorax 

Hemothorax, an exceedingly rare complication of CIED placement, is primarily linked to lead perforation into the right ventricle and pericardium, leading to intrusion into the pericardial and then pleural space. Additionally, it can lead to rise trauma to adjacent vascular structures or direct lung injury. In exceptionally rare cases, hemothorax during lead insertion may occur due to intercostal vessel rupture, leading to profuse bleeding triggered by forceful coughing induced by a rapid increase in pleural pressure. It is imperative never to cannulate the artery with the introducer, as this situation necessitates immediate vascular surgical intervention. To prevent such occurrences, it is crucial to consistently verify the fluoroscopic path of the guidewire into the inferior vena cava before introducer insertion [11].

### 3.3 Pulmonary Air Embolism

Pulmonary air embolism represents a critical complication when gas enters the pulmonary artery via the venous route, typically through the right heart chambers. This condition is exclusively iatrogenic and commonly associated with central venous access procedures.

During deep inspiration, negative intrathoracic pressure is generated, predisposing to significant air influx into the venous system during central venous access. Operator expertise and using hemostatic valves with introducers are pivotal in preventing this complication. Operators must be vigilant for the characteristic hissing sound, indicative of air entry, which can be further confirmed with fluoroscopy [12].

Clinical manifestations of pulmonary air embolism range from asymptomatic cases to respiratory distress, hypotension, and desaturation, contingent upon embolus size. Management typically involves administering 100% oxygen, while vasopressors or inotropics may be necessary in severe cases. Fortunately, the embolism resolves spontaneously as the lungs filter and absorb the air.

## 4. Lead-Related Complication

### 4.1 Cardiac Perforation

**Fig. 2.**
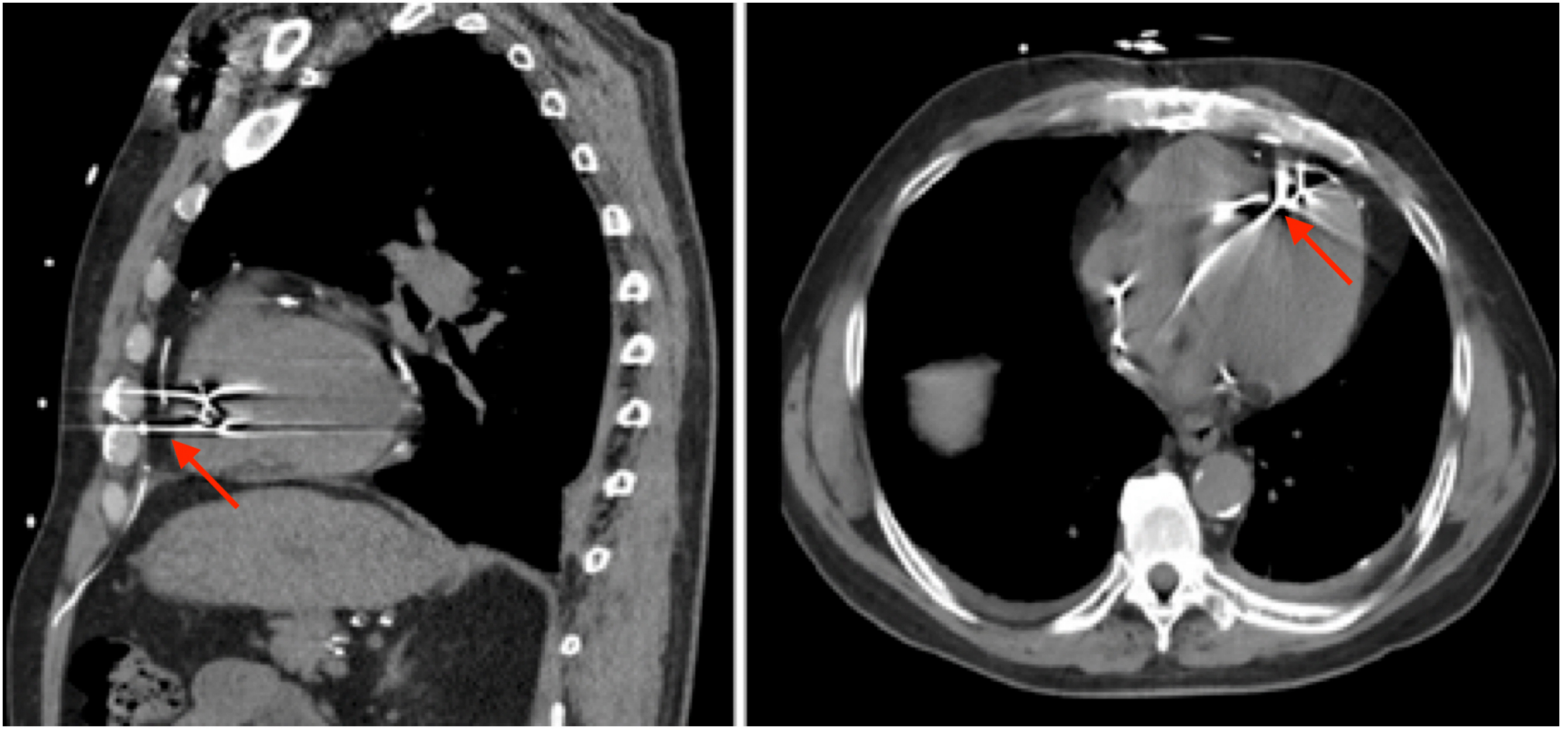
**Cardiac perforation**. Lead tip perforating the right ventricular myocardium and pericardium (Red arrow). Simsolo, E., & Wilkoff, B. L. (2022). [Image]. Open Access Article. Retrieved from https://www.jacc.org/doi/10.1016/j.jaccas.2022.07.003[13].

**Fig. 3.**
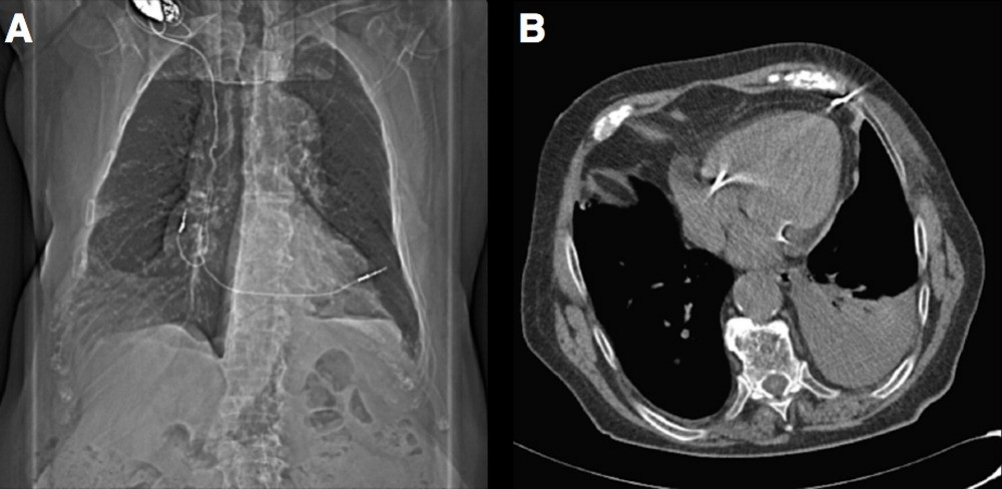
**Cardiac perforation**. (A) Chest computed tomography showing lead perforation. (B) Computed tomography showing lead anchorage to chest wall. Prestipino, F., Nenna, A., Casacalenda, A., & Chello, M. (2014). [Image]. Creative Commons license. Retrieved from https://www.sciencedirect.com/science/article/pii/S2210261214002545[14].

Myocardial perforation remains a rare, yet significant complication associated with pacemaker lead insertion and extraction procedures. It occurs primarily due to manipulation of the lead or fixation screw, resulting in bleeding into the pericardial space. This complication can affect various parts of the heart that come into contact with the lead, including the great veins, atria, or ventricles (Fig. 2, Ref. [13]; Fig. 3, Ref. [14]). The right ventricular apex is the most common site for perforation due to its thinner myocardial wall [15].

Perforation can be acute (within 24 hours after implantation), sub-acute (between 24 hours and one month after implantation), or chronic (occurring more than one month after implantation). Symptoms of lead perforation may include acute chest pain with or without hypotension, along with significant periprocedural complications such as pericardial effusion. Regardless of the timing of insertion, any changes in lead parameters and pericardial effusion observed on imaging modalities such as transthoracic echocardiography (TTE), CT, magnetic resonance imaging (MRI), or diaphragmatic stimulation during right ventricular bipolar pacing should raise suspicion of cardiac perforation.

While perforation typically occurs without serious consequences, it can lead to cardiac tamponade, necessitating immediate diagnosis, hemodynamic resuscitation with volume and pressors, and interventions such as percutaneous pericardiocentesis. In some cases, surgical intervention may be required if bleeding persists. Fluoroscopy of the left heart border showing a lack of movement can provide an initial indication of bleeding, which TTE can further confirm. Other signs pointing towards perforation include poor implant pacing threshold and disparities in unipolar versus bipolar sensing.

Rarely, during lead extraction procedures, direct bleeding into the mediastinal space can occur due to trauma to the great veins above the pericardial reflection. This can have fatal consequences and warrants emergent surgical intervention. Early recognition and prompt management of myocardial perforation are crucial in preventing severe complications and ensuring favorable patient outcomes.

### 4.2 Venous Thrombosis

Significant venous thrombosis of the innominate or subclavian veins, with near-total occlusion, is one of the complications associated with lead implantation. Difficulty in gaining access during the initial implantation, coupled with venous injury from trauma and subsequent inflammation, serves as the primary trigger. This spectrum of complications may range from asymptomatic cases detected during revision procedures to even manifesting as superior vena cava syndrome (SVCS).

Symptoms include arm swelling, collateral veins on the arm, thorax, or abdomen, and possible associated facial suffusion, cyanosis, or edema with head and neck discomfort.

Predictive factors for the occurrence of SVCS have been consistently elusive, with variable associations with the

(1) Total number of leads

(2) Are the leads intact or severed?

(3) Timing since the initial device implant

(4) Ongoing infection, i.e., endocarditis

Infection should almost always be a differential diagnosis considered in these cases. Literature suggests no difference in the incidence of Venous thrombosis when comparing the cephalic cutdown approach to the subclavian puncture technique and the use of lead insulation type.

Management depends on whether thrombosis or fibrosis is causative and varies from heparin followed by warfarin or thrombolysis to percutaneous angioplasty or open surgical procedures. Early vascular specialist consultation should be sought. In summary, venous abnormalities occur frequently, but they are rarely of clinical significance.

A rare associated complication is pulmonary thromboembolism, which is potentially life-threatening. The presence of pulmonary embolism in a patient with a device should raise suspicion of thrombotic lead source.

### 4.3 Lead Dislodgment

**Fig. 4.**
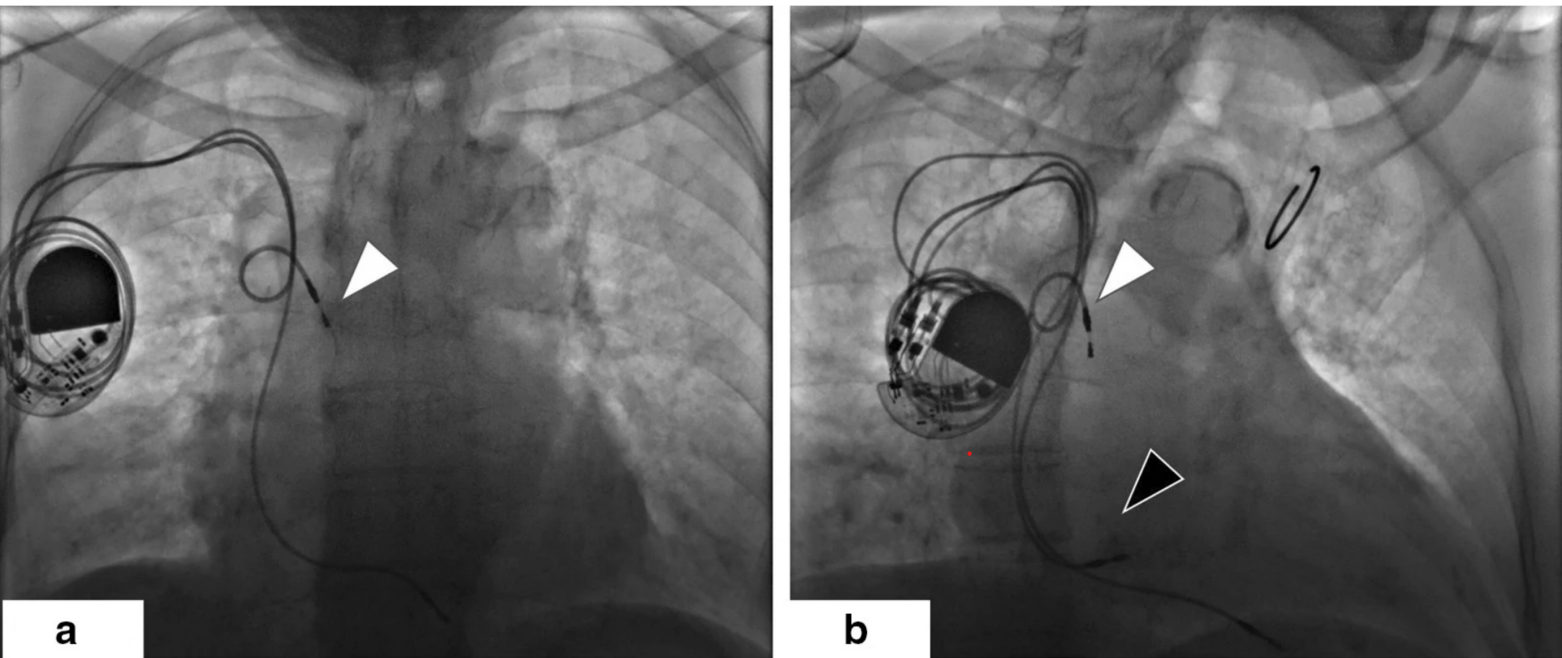
**Anteroposterior projected radiograph of the patient before (a) and after (b) new atrial lead implantation**. (a) The dislodged atrial lead in the superior vena cava, indicated by the white arrow. (b) The new atrial lead implanted in the right lower septum, indicated by the black arrow. Guan, F., Li, G., Liu, Y., Gao, X., & Zhou, R. (2020). [Image]. Open Access Article. Retrieved from https://jmedicalcasereports.biomedcentral.com/articles/10.1186/s13256-020-02626-z#auth-Fu-Guan-Aff1[16].

Lead dislodgment, defined as any evidence of electrical or imaging suggesting displacement of the lead from its original implant site, is one of the most common complications of pacemaker (PPM) procedures (Fig. 4, Ref. [16]). It typically occurs very early post-procedure (within 1–2 days) but may also manifest up to 120 days after initial implantation.

Intermittent undersensing or loss of capture following a PPM procedure is a strong indication for further evaluation with device interrogation. Imaging modalities such as chest X-rays can occasionally reveal evidence of macro-dislodgment [17].

Dislodgment of the right ventricular pacing lead can fail to capture, potentially leading to syncope or even sudden death in patients with unstable underlying rhythms. Atrial lead dislodgement may cause inappropriate atrial or ventricular arrhythmias detection, leading to atrioventricular dyssynchrony. Dislodgment of left ventricular (LV) pacing leads to CRT, which can result in ventricular dyssynchrony and worsening heart failure. LV leads, in particular, have a higher incidence of dislodgment than atrial or right ventricular leads. Active fixation leads have been associated with reduced events of LV lead dislodgment compared to passive fixation, as indicated in the literature.

To mitigate the risk of dislodgment, it is imperative to identify potential risk factors during device implantation. Strategies may involve monitoring fluoroscopic stability while exerting forward pressure, assessing lead redundancy, and evaluating loss of capture while pacing just above the capture threshold, particularly in scenarios inducing negative intrathoracic pressure, such as deep inspiration. Previous research has highlighted female sex and higher body mass index (BMI) as risk factors linked to increased lead dislodgment or displacement. The greater variation in lead tension post-implantation due to adipose tissue in obese female patients heightens the dislodgment risk. Hence, operators should allow more slack in the leads during implantation and verify lead positioning during deep inspiration. Employing meticulous closure techniques, including precise suturing of the device and lead sleeves to the fascia, also serves as crucial preventive measures.

Studies indicate a higher likelihood of recurrent dislodgment in patients who undergo repositioning of previously dislodged leads compared to replacement with new leads. Potential explanations include inherent flaws in lead deployment or myocardial tissue damage hindering reattachment at the lead tip. Early detection and proactive prevention strategies are vital for minimizing the risk of lead dislodgment and enhancing patient outcomes in PPM procedures.

### 4.4 Conductor Fracture

**Fig. 5.**
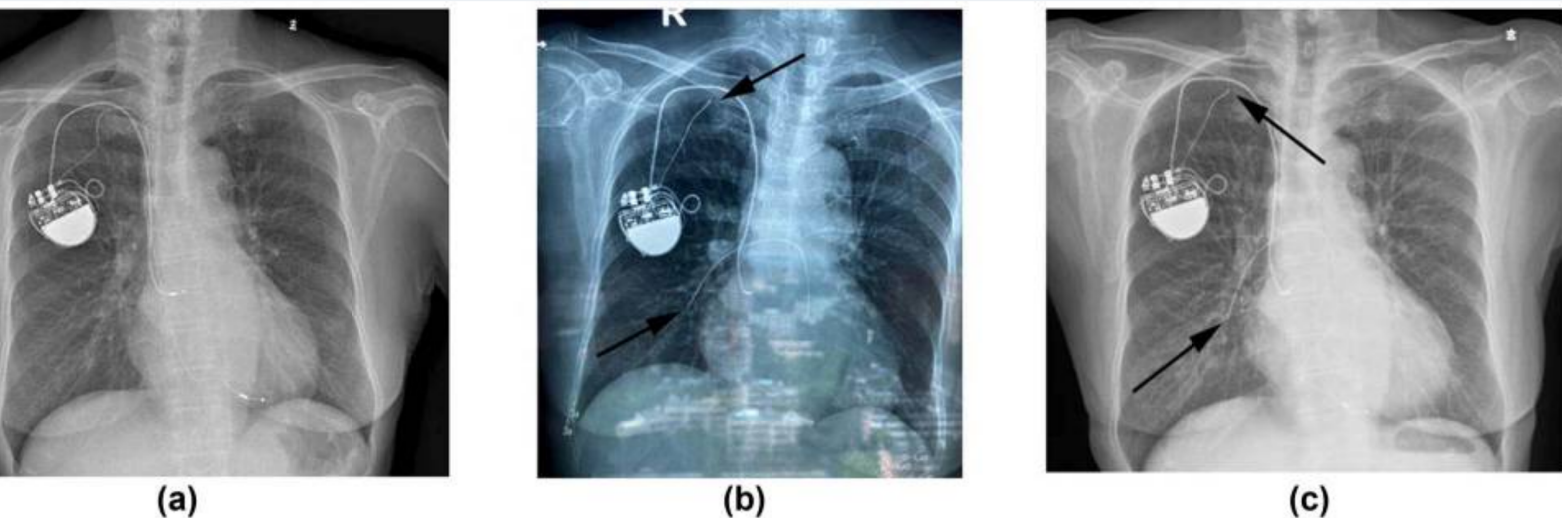
**Chest X-ray images (a) showing both leads were intact, while (b) and (c) showing a complete right-ventricular lead fracture (Black Arrow)**. Xu, Y., Chen, X., Feng, J., Guo, J., & Li, Z. (2021). [Image]. Creative Commons Attribution 4.0 International License. Retrieved from https://www.ncbi.nlm.nih.gov/pmc/articles/PMC8491585/[18].

Conductor fracture denotes interruptions in the wire component of the lead. In contrast to ICDs, pacing/sensing thresholds are usually not significantly impacted in this situation owing to the lower current used in pacing, although pacing impedance may rise. Identifying a pacemaker lead fracture on a chest X-ray involves recognizing specific signs that suggest a discontinuity or abnormality in the course of the pacemaker lead (Fig. 5, Ref. [18]). A conductor fracture contributes impedance in series with the circuit, while an insulation breach introduces a secondary current pathway in parallel with the circuit. Theoretically, impedance increases with a conductor fracture and decreases with an insulation breach. Nevertheless, in clinical practice, impedance often remains within the normal range at initial suspicion or diagnosis of fractures and insulation breaches.

### 4.5 Insulation Failure

Insulation failure refers to damage to the polymer covering the conductor. Such damage can occur during the procedure due to instrument manipulation or tight tie-down sutures, or it may gradually develop over time due to friction against the device. When insulation fractures occur, pacing impedance is notably reduced. Differentiating between inner and outer insulation or conductor breach can be achieved by examining pacing impedance in the unipolar pacing mode within a bipolar lead system.

Lead damage often results from a puncture site in the medial subclavian vein during initial implantation, allowing compression of the lead by the first rib, clavicle, and surrounding structures. Strategies to prevent conductor and insulation failure include utilizing a far lateral subclavian vein insertion or cephalic cutdown approach, advising patients to refrain from heavy upper limb exercises, and employing smaller diameter leads. By implementing these preventive measures, the occurrence of conduction fractures and insulation breaches can be minimized, thus optimizing the performance and lifespan of cardiac leads.

### 4.6 Pacemaker Exit Block

Pacemaker exit block refers to the failure or delay of a pacemaker impulse to discharge the surrounding myocardium. Although increasingly rare, it remains an important complication of pacemaker implantation. Initially, it may be asymptomatic but can be detected by slowly increasing pacing thresholds and changes in impedance, particularly in the absence of antecedent trauma or lead fracture.

The mechanism of the pacemaker exit block typically involves scar tissue formation or calcium crystal deposition, which impedes the transmission of pacing impulses. This complication can be life-threatening, especially in patients who are pacing dependent and may necessitate leading repositioning or replacement.

The incidence of pacemaker exit blocks has significantly decreased with modern pacing leads, particularly those with steroid-eluting tips. These leads help mitigate tissue reaction and scarring, reducing the likelihood of exit block occurrence.

Early recognition of pacemaker exit block is essential for timely intervention to prevent adverse outcomes. Close monitoring of pacing thresholds and impedance, along with regular follow-up appointments, can aid in detecting this complication. Prompt action, such as lead repositioning or replacement, may be necessary to ensure optimal pacemaker function and patient safety.

### 4.7 Connection Problem

Older generation leads frequently exhibited susceptibility to various types of connection problems. However, with newer generation leads, the most common issues encountered are incomplete pin insertion into the header or header-lead pin mismatch.

Header-lead pin mismatch can result in abrupt increases in subthreshold impedance measurements, especially in headers utilizing canted-coil spring contacts. Friction from minor movements of the ring electrode within the header can produce microscopic particles that oxidize, thereby escalating the impedance between the spring contact and ring. Diagnosis of header-lead pin mismatch necessitates the exclusion of other causes, typically confirmed through radiography or visual inspection showing complete pin insertion, absence of overspending, and no alteration in pacing threshold.

Failure to adequately seat the proximal lead connection into the generator header may seem trivial, but it does occur. In this situation, the inadequate connection may generate electrical noise with consequent oversensing or a make-break connection problem with under sensing or failure to pace [19].

Rarely, issues such as loose set screws, setscrew misalignment, and failure of the adhesive bonding the header to the generator may also be observed.

### 4.8 Connection Problems vs Insulation/Conductor Issues

Distinguishing between various connection problems and insulation/conductor fractures involves considering several critical factors. In the realm of connection issues, a notable rise in unipolar-tip or integrated-bipolar impedance indicates a probable conductor fracture at the tip electrode. While conductor fractures are a rarity within the first-year post-lead implantation, connection problems typically surface earlier, soon after the lead and generator connection. Loose set screws are commonly detected peri-operatively, whereas incomplete pin insertion might manifest within the initial six months or even later. As time progresses, setscrew misalignment and header-bond failure may emerge. The absence of oversensing leans towards a connection problem, particularly if noticeable over an extended period (e.g., one month) post-impedance rise. Notably, in defibrillation-4 (DF-4) leads, incomplete pin insertion impacts all conductors, excluding incomplete pin insertion as the solitary cause if there’s a sudden increase solely in pacing impedance.

Understanding these distinguishing features is crucial in accurately diagnosing and managing various connection problems in pacemaker leads, thereby ensuring optimal device performance and patient outcomes [20].

### 4.9 Device Related Infection

Infection of the subcutaneous pocket surrounding CIEDs can occur during implantation or subsequent manipulations, such as generator changes. Sometimes, the generator or subcutaneous electrodes may erode through the skin, increasing the risk of Infection.

Over the past decade, there has been a concerning trend of increasing infection burden associated with pacemaker implantations. This trend can be attributed to the rise in comorbidities among patients, including renal failure, heart failure, diabetes mellitus, and chronic obstructive pulmonary disease (COPD). Higher infection rates have been linked to longer hospital stays, increased healthcare costs, and higher mortality rates.

Factors contributing to the increased risk of Infection include preprocedural temporary pacing, lack of periprocedural antimicrobial prophylaxis, and early reintervention following a procedure. The Infection can spread from the pocket to the intracardiac portion across the leads or through hematogenous spread from the leads or pacemaker pocket itself.

Symptoms and signs of pocket site infection include pain, erythema, warmth, swelling, ulceration, and drainage. Systemic infections such as endocarditis or osteomyelitis may manifest with fever and chills. Laboratory studies, including blood cultures, complete blood counts, and C-reactive protein levels, are essential for diagnosis. Normal results for these investigations do not rule out CIED Infection. Empiric treatment typically involves vancomycin or daptomycin, with the duration of treatment ranging from 1 to 6 weeks, depending on the type of Infection (Table 3).

**Table 3.** **Pacemaker infections**.

Symptoms	
	Fever
	Pain, redness, or swelling at the implantation site
	Malaise or fatigue
	Chills
Clinical findings	
	Assess vital signs
	Evaluate pacemaker site for signs of infection
	Obtain patient history, including recent procedures or surgeries
Diagnostic tests	
	Blood culture
	Echocardiogram (TTE/TEE)
	Complete blood count (CBC) with differential
	C-reactive protein (CRP) level
	Erythrocyte sedimentation rate (ESR)
Indicators for infection	
	Positive blood culture indicating bacteremia
	Evidence of vegetation on echocardiogram (TTE/TEE)
	Elevated inflammatory markers (CRP, ESR)
Treatment	
	Empirical antibiotic therapy pending culture results
	Surgical intervention may be necessary for device removal in severe cases
	Tailor antibiotic regimen based on culture and sensitivity results
	Monitor for complications such as endocarditis or septicemia

TTE, transthoracic echocardiography; TEE, transesophageal echocardiography.

Studies have shown a significantly reduced infection rate with 1 g of cefazolin immediately before the procedure. This finding is reinforced by the American Heart Association (AHA), which recommends antibiotic administration within 1 hour of incision for CIED implantation as a Class IA recommendation. Additionally, AHA guidelines advocate for preoperative antiseptic preparation and general intraprocedural sterile precautions. Perioperative skin preparation with chlorhexidine-alcohol is preferred over povidone-iodine in reducing surgical site infections.

Adhering to these recommendations and implementing effective infection prevention measures can mitigate the incidence of CIED-related infections, improving patient outcomes and reducing healthcare burdens [21].

### 4.10 Pocket Hematoma

Pocket hematoma is one of the most common complications following pacemaker implantation. While mostly benign, it can increase the risk of prolonged hospitalization, reoperation, and device-related infections. Proper patient preparation, attention to modifiable risk factors, and operator experience are crucial for preventing pocket hematoma.

Risk factors for pocket hematoma include antiplatelet therapy, device replacement, lead revision, and heparin bridging. Therefore, optimizing antiplatelet/anticoagulant regimens before pacemaker implantation is necessary for prevention. Studies have identified pre-procedural receipt of dual antiplatelet or heparin/low-molecular-weight heparin (LMWH) products as significant contributing factors to pocket hematoma formation. Similarly, a randomized control trial demonstrated that intravenous heparin administration significantly increased hematoma formation compared to patients who did not receive intravenous (IV) heparin. Post-CIED implantation LMWH is also associated with a significant increase in pocket hematomas [22].

Given the identified risk factors, optimization of anticoagulant regimens before CIED implantation is essential for safe patient care. For patients at low thromboembolic risk, withholding warfarin for a few days before implantation may be suitable. Conversely, for patients at moderate to high thromboembolic risk, oral anticoagulants should be continued during implantation, with efforts made to avoid bridging therapy. By understanding these risk factors and implementing appropriate anticoagulant strategies, healthcare providers can reduce the incidence of pocket hematoma following pacemaker implantation, ultimately improving patient outcomes.

### 4.11 Twiddler’s Syndrome

During the initial phase following implantation, patients commonly engage in adjusting the implanted device as an instinctual response to alleviate discomfort arising from the healing process or to confirm the functionality of the device, adapting to the presence of this foreign object [23]. This tendency tends to diminish swiftly among patients devoid of prior psychiatric conditions and possessing a heightened awareness of their medical condition. Conversely, individuals with psychiatric ailments or limited awareness of their condition often persist in manipulating the device. It is imperative to promptly identify such behavior to forestall displacement of the device and intervene decisively. Montisci *et al*. [24] in his study recommends integrating a comprehensive biopsychosocial approach into clinical practice, incorporating psychiatric assessments for patients undergoing CIED implantation. This proactive approach can aid in identifying individuals prone to device manipulation, guiding clinicians in selecting suitable device types, implementing surgical techniques to mitigate manipulation risks, or potentially initiating psychiatric interventions.

### 4.12 Electromagnetic Interference

Though the potential for electromagnetic interference with pacemakers and cardiac resynchronization devices in nonclinical environments exists, the probability of encountering a significant issue is exceedingly low. Manufacturers of pacemakers do not recommend any specific precautions when using common household appliances provided, they are in good working condition. Patients with pacemakers should be cautious around strong magnetic fields, as they can disrupt the normal operation of cardiac devices. Smartphones lacking strong magnets are unlikely to cause notable interference with pacemakers or ICDs. It’s advisable for all patients to use cell phones on the opposite side of the head from where the cardiac device is located and to carry phones in a pocket below the waist. While prolonged exposure to electromagnetic security systems has been linked to pacemaker inhibition, such occurrences are rare with brief exposure. Patients should be made aware of the presence of security systems and encouraged to pass through them at a regular pace.

Numerous patients undergoing continuous flow left ventricular assist device implantation also possess other concurrent implantable cardiac devices. While these devices typically operate effectively, there exists a risk of electromagnetic interference (EMI). Such interference may result in challenges with telemetry establishment and compromised electrical signal sensing [25]. The nature of interference, as well as treatment approaches, varied and depended on the specific device involved. Techniques employed to mitigate interference included the utilization of metal shielding, physical adjustments to increase the distance between devices, and in some cases, replacement of the implanted device with an alternative device within the same category. To prevent future occurrences of EMI, it is imperative for physicians to remain cognizant of documented instances of interference between specific devices, and for manufacturers to collaborate more closely to enhance the compatibility of implanted cardiac devices.

### 4.13 Complication with Leadless Pacemaker Technology

The introduction of leadless pacemakers represents a significant technological advancement, although their effectiveness remains uncertain based on current evidence. Unlike traditional models that rely on leads attached to the heart muscle for stability, leadless pacemakers are autonomous devices implanted directly into heart tissue. This innovative approach in cardiac pacing aims to address the limitations and challenges of conventional transvenous pacemakers, including device-related complications [26]. Despite their advantages such as compact size, less invasive implantation, and reduced risk of lead-related problems, pneumothorax, and endocarditis, leadless pacemakers still carry potential complications.

With transvenous pacemakers, there is a higher incidence of endocarditis compared to leadless pacemakers, often necessitating lead removal. The absence of implanted leads may provide benefits in reducing the risk of bloodstream infection and endocarditis, as there is a lower potential for:

(i) Biofilm accumulation,

(ii) Thrombus formation (which can lead to complications such as stroke or systemic embolism),

(iii) Disruptions in flow dynamics,

(iv) Interference with heart valves [27].

A significant concern revolves around the risk of leadless pacemaker displacement or migration. Despite being designed for secure anchorage within the heart, there remains a possibility of positional shifts over time, potentially resulting in inadequate pacing or even heart muscle perforation. Additionally, the implantation procedure itself can lead to complications associated with leadless pacemakers. Although less invasive compared to traditional methods, the procedure still entails risks such as bleeding, infection, and potential damage to adjacent structures.

In a meta-analysis by Shtembari *et al*. [28] comparing the safety of leadless pacemakers (LP) and transvenous pacemakers (TVP) demonstrated a 42% lower overall odds of complications for patients with LPs in comparison to TVPs. More specifically, the odds of device dislodgment and the need for re-intervention were reduced by 70% and 46%, respectively. The occurrence of pneumothorax was also notably lower, with an 87% reduction in odds. However, this analysis also highlighted a potential concern with LPs: a more than two-fold increase in the odds of pericardial effusion. Adding to the concerns, a study by Piccini *et al*. [29] found that patients who received the Micra leadless pacemaker were twice as likely to experience a perforation/effusion event compared to those who received a transvenous pacemaker. These findings suggest a generally favorable safety profile for LPs, although the increased risk of pericardial effusion warrants careful consideration. The study underscored the need for randomized controlled trials to further elucidate these findings, given the observational nature of the data reviewed. Furthermore, due to the specialized delivery systems and implantation techniques required for leadless pacemakers, there is a learning curve for physicians, potentially increasing the risk of procedural complications, especially in the early stages of adoption.

## 5. Conclusions

In conclusion, advancements in CIEDs have revolutionized cardiac care, offering patients increasingly sophisticated options for managing cardiac rate and rhythm. However, along with these innovations come a spectrum of potential complications that clinicians must navigate. From access-related issues like pneumothorax and hemothorax to lead-related complications such as dislodgment and conduction fractures, each complication presents unique challenges that require careful management and proactive prevention strategies.

Additionally, device-related infections and pocket hematomas further underscore the importance of meticulous perioperative care and Infection prevention measures. By understanding the nuances of these complications and implementing evidence-based strategies for prevention and management, healthcare providers can optimize patient outcomes and enhance the safety and efficacy of CIED implantation procedures.

Continued research and technological advancements will further refine our approach to managing these complications, ultimately improving the quality of care for patients requiring cardiac pacing. While the leadless pacemakers offer several benefits over conventional counterparts, such as a decreased risk of lead-related issues and a smaller implant profile, they are not without potential complications. Vigilant monitoring and careful patient selection are essential to mitigate the risks associated with leadless pacemaker implantation and ensure favorable outcomes for individuals with cardiac rhythm disorders.
